# Periprocedural Strategies for Stroke Prevention in Patients Undergoing Transcatheter Aortic Valve Implantation

**DOI:** 10.3389/fcvm.2022.892956

**Published:** 2022-04-26

**Authors:** Matthias Linder, Moritz Seiffert

**Affiliations:** ^1^Department of Cardiology, University Heart & Vascular Center Hamburg, University Medical Center Hamburg-Eppendorf, Hamburg, Germany; ^2^German Centre for Cardiovascular Research (DZHK), Partner Site Hamburg/Lübeck/Kiel, Hamburg, Germany

**Keywords:** anticoagulation, antithrombotic therapy, cerebrovascular embolic protection, stroke, TAVI

## Abstract

Cerebrovascular events remain a serious complication in patients undergoing transcatheter aortic valve implantation with an incidence of 2–3% at 30 days. While expanding TAVI to younger low-risk patients, prevention of periprocedural strokes becomes even more important. Different cerebral embolic protection devices have been tested but a clear clinical benefit has not been demonstrated in randomized trials. Due to the multifactorial aetiology with different predisposing factors, stroke prevention should include procedural and periprocedural strategies. This article aims to summarize different approaches and discuss open questions.

## Cerebrovascular Events in Patients Undergoing TAVI

**FIGURE 1:**
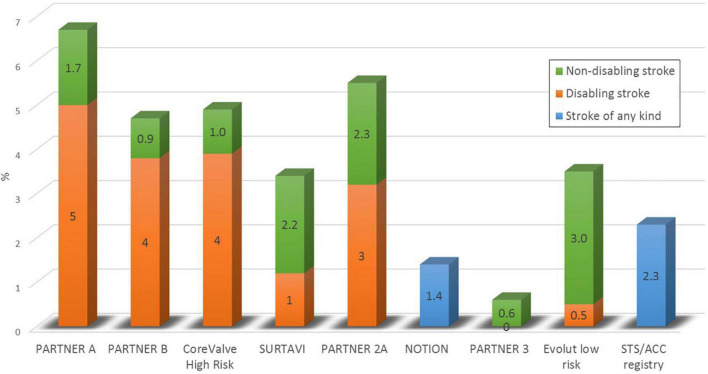
The incidence of stroke within 30 days after TAVI in randomized controlled trials and in a real world clinical registry.

Despite continuous improvements in outcomes after transcatheter aortic valve implantation (TAVI), periprocedural strokes remain a devastating complication with severe implications on quality of life, morbidity, and mortality (1, 2). Large trials demonstrated a reduction in periprocedural complications and mortality in the past years, most probably due to procedural and device-related refinements and the expansion of TAVI to younger low-risk patients (3–8). Subsequently, the PARTNER 3 trial reported an incidence of only 0.6% for strokes within 30 days after TAVI in a highly selected low-risk patient cohort (8) (see Table 1 and Figure 1). But the applicability of these encouraging results to an all-comers patient population remains to be determined. A recent analysis of more than 100,000 patients from the Society of Thoracic Surgeons/American College of Cardiology Transcatheter Valve Therapies Registry reported an overall stroke rate of 2.3% after TAVI in a real-world patient population which remained largely unchanged from 2011 to 2017 (9). Predictors for stroke after TAVI have frequently been investigated with varying and partly conflicting results, underlining the multifactorial aetiology of cerebrovascular events. These included female sex, chronic kidney disease, impaired left-ventricular function, bicuspid aortic valves, aortic stenosis severity, a history of stroke, atrial fibrillation and higher CHA2DS2-Vasc scores, and spontaneous echo contrast in the left atrial appendage for patient-related factors. Procedure-related risk factors were non-transfemoral access, embolization and migration of the transcatheter heart valve, prolonged procedure duration or

**TABLE 1:** Incidence of stroke events at 30 days after TAVI in randomized controlled trials and registries.

Name of study	Number of patients	Age	STS-PROM	Non-disabling stroke	Disabling stroke	Any stroke
PARTNER A	179	83.9	11.2%	1.7%	5.0%	6.7%
PARTNER B	348	83.6	11.8%	0.9%	3.8%	4.7%
CoreValve High Risk	390	83.2	7.3	1.0%	3.9%	4.9%
SURTAVI	864	79.9	4.4%	2.2	1.2%	3.4%
NOTION	145	79.9	2.9%	n/a	n/a	1.4%
PARTNER 2A	1011	81.5	5.8%	2.3%	3.2	5.5%
PARTNER 3	496	73.3	1.9%	0.6%	0.0%	0.6%
Evolut low risk	725	74.1	1.9%	3.0%	0.5%	3.4%
STS/ACC registry	101 430	83.0	6.0%	n/a	n/a	2.3%

balloon postdilatation. Postprocedural aspects included antithrombotic medication or new-onset atrial fibrillation (1, 10–14) (see Figure 2).

**FIGURE 2:**
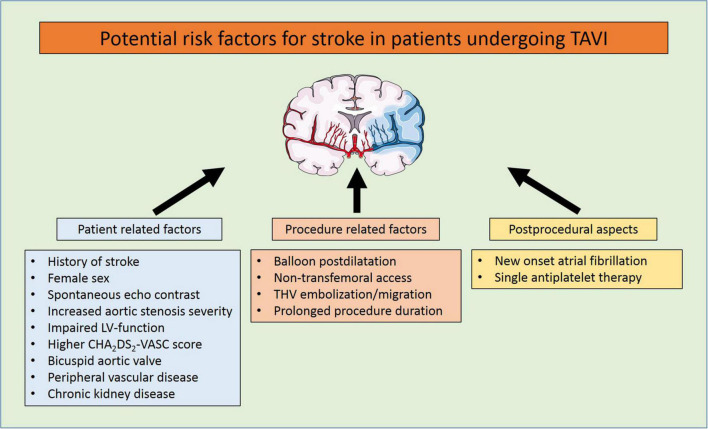
Potential risk factors for stroke in patients undergoing TAVI.

## Timing and Characterization of Periprocedural Strokes

**FIGURE 3:**
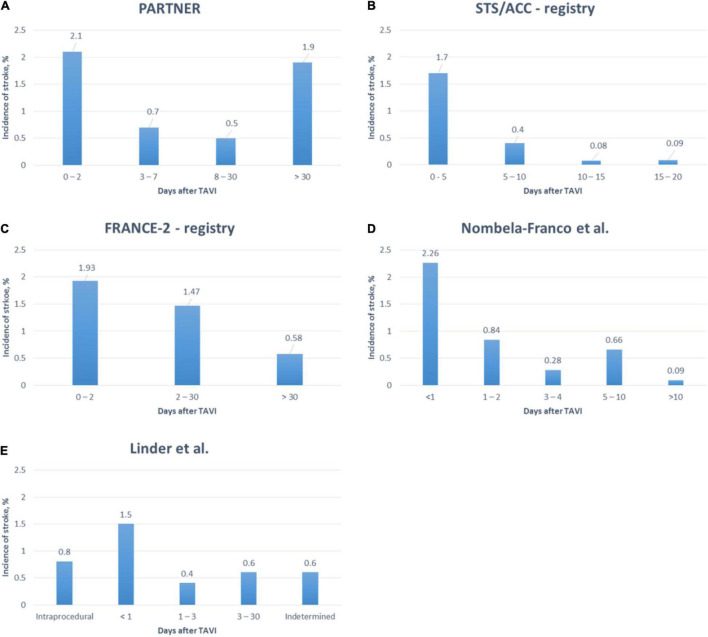
Timing of stroke after TAVI in a randomized controlled trial (18) and several registries (9, 15–17).

According to several studies, 91.5–98.3% of cerebrovascular events were of ischemic origin and most strokes became apparent within the first days following TAVI (9, 15–18). However, stroke did not necessarily occur during the procedure itself (see Figure 3). Pathology studies identified thrombus, aortic valve tissue, foreign materials, calcium, endothelium, myocardium, and collagen as captured debris in CEP (19). Transcranial Doppler studies detected high-intensity transient signals (HITS) in every TAVI procedure, predominantly during manipulation of the calcified aortic valve during THV positioning and implantation (20, 21). However, the association of HITS and clinically apparent strokes remains to be determined at current. Furthermore, several studies investigated brain lesions after TAVI on DW-MRI (17). Most of these lead to silent brain infarctions (SBI) rather than to clinically apparent strokes. SBI were linked to cognitive decline, perioperative delirium and a composite of overt strokes or TIA after non-cardiac surgery (18). A recently published meta-analysis found SBI in at least 70% of patients following TAVI and linked it to impaired early neurocognitive outcomes (19). The long-term impact of SBI after TAVI remains yet to be determined.

## Cerebral Embolic Protection Devices

While expanding TAVI to younger and lower risk patients, procedural safety—including the prevention of periprocedural strokes in particular—becomes even more important. One approach to reduce procedural cerebrovascular events is to capture or deflect embolic debris from the brain-supplying arteries during TAVI. Several devices for cerebral embolic protection (CEP) have been designed and a significant increase of CEP utilization has recently been described for the United States (2017: 2.8%, 2018: 17.3%) (22). Three devices, that are currently used in clinical routine or under clinical investigation, are discussed briefly:

### Sentinel

**FIGURE 4:**
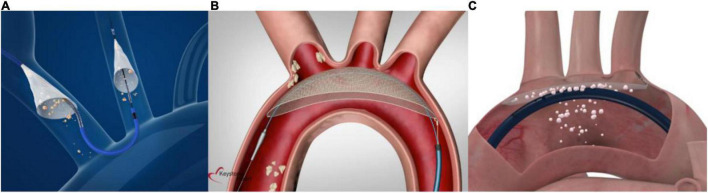
Current cerebral embolic protection devices. **(A)** The Sentinel device is a dual- filter-based intra-luminal embolic protection device introduced via the right radial, ulnar, or brachial artery. A proximal filter is placed in the brachiocephalic trunk and a distal filter in the left carotid artery (material provided courtesy of Boston Scientific.© 2022 Boston Scientific Corporation or its affiliates. All rights reserved). **(B)** The TriGuard3 device is advanced to the aortic arch via the contralateral femoral artery and accommodates a diagnostic pigtail catheter. It covers all three supraaortic vessels to deflect embolic material (material provided courtesy of Keystone Heart, Ltd. All rights reserved). **(C)** The ProtEmbo device is delivered via the left radial arteria and deployed at the aortic arch roof to cover all three supraaortic vessels and deflect embolic material (material provided courtesy of Protembis GmbH. All rights reserved).

The SentinelTM (Boston Scientific, Marlborough, Massachusetts) is a CE-marked, FDA-approved, and widely commercially available CEP. It consists of a 6-French compatible dual-filter (140 μm pore size) intra-luminal embolic protection device introduced via the right radial, ulnar, or brachial artery. A proximal filter is positioned in the brachiocephalic artery and a distal filter is deployed in the left carotid artery for the time of the TAVI procedure (see Figure 4A), as previously described (23). At the end of the procedure, both filters are withdrawn.

Several studies demonstrated a reduction in the total volume of new cerebral lesions, however, no significant changes in the number of patients with new lesions or the total number of new lesions were observed (23–25). In addition, these trials failed to demonstrate a significant reduction of clinically apparent stroke events due to CEP in patients undergoing TAVI (26). On the contrary, a pooled propensity matched pair analysis of randomized and non-randomized linked the SentinelTM CEP to lower rates of strokes within 72 h and mortality at 30 days (27). As results were largely driven by an unusually high stroke rate in the control group of the non-randomized patient population, these findings should be interpreted with caution.

The left vertebral artery arising from the left subclavian artery remains unprotected with the SentinelTM. This may leave a relevant number of patients only partially protected against embolic debris and illustrates a potential limitation of a dual filter CEP-device (15).

To gain additional evidence on the reduction of clinically apparent strokes with CEP during TAVI, the large-scale PROTECTED TAVR (NCT04149535) and British Heart Foundation PROTECT-TAVI (ISRCTN16665769) trials are underway and first results are expected to be presented soon.

### TriGUARD3

The TriGUARD3 (Keystone Heart Ltd., Tampa, Florida) is the latest iteration of a 8-French compatible self-positioning deflection filter system (115 μm pore size) that is introduced through the common femoral artery and deployed in the aortic arch (see Figure 4B). By covering all three major cerebral vessels during TAVI, embolic debris is deflected into the descending aorta. The device lumen allows for the placement of a pigtail catheter into the aortic root to guide the TAVI procedure (28). The device received CE-mark in 2020.

After evaluation of the earlier TriGuard™ CEP in the DEFLECT III trial (29), the results of REFLECT II for the TriGUARD3 were recently presented (28). This study was designed to evaluate safety and efficacy of the TriGUARD3 CEP in reducing clinical events and cerebral lesions during TAVI. The trial was prematurely terminated by the sponsor at recommendations of the FDA and Data and Safety Monitoring Board after enrollment of 179 of 225 planned patients. Compared to a historical performance goal, the REFLECT II trial demonstrated the use of the TriGUARD3 to be safe and met the composite 30-day primary safety endpoint according to VARC-2 (30). However, the trial failed to demonstrate efficacy compared with the controls in the pre-specified superiority primary hierarchical composite of all death or stroke at 30 days, NIHSS score worsening, freedom from cerebral ischemic lesions, and total cerebral lesion volume (28). Of note, interaction with the transcatheter heart valve delivery system was reported in 9.6% of cases and complete cerebral coverage was achieved in only 59.7% of patients according to the core laboratory analysis. If complete coverage in all patients may have altered clinical efficacy remains unclear at present.

### ProtEmbo

The ProtEmbo Cerebral Protection System (Protembis GmbH, Aachen, Germany) is currently under clinical investigation. It consists of a 6-French compatible implantable aortic filter (pore size 60 μm) designed to cover all three major cerebral arteries in the aortic arch and deflect embolic debris away from the cerebral circulation into the descending aorta during TAVI. It is delivered via the left radial arteria, deployed at the aortic arch roof and retrieved at the end of the procedure (see Figure 4C). A first in human use (31) was the very first case of the PROTEMBO C trial, a European multi-center, single-arm trial evaluating the safety and efficacy in 60 patients undergoing TAVI (NCT04618718). Results of the study are expected to be presented shortly.

Despite promising results in smaller single-center analyses for CEP devices, trials have failed to decrease stroke rates in randomized controlled trials until now. Due to an unclear clinical benefit and additional economic constraints, widespread clinical adoption remains limited at current.

## Additional Strategies for Stroke Prevention

The observation that a relevant number of strokes occur early after TAVI but not necessarily during the procedure itself (9, 15–18) and that no CEP has yet demonstrated a significant reduction in stroke rate suggests that a sufficient CEP strategy should expand “beyond the procedure itself.” In addition to refined CEP devices, this may include a tailored pharmacological approach to prevent peri- and postprocedural cerebrovascular events by targeting risk factors. Anticoagulation and antithrombotic therapies are a mainstay of this approach.

### Periprocedural Anticoagulation

In the absence of specific recommendations for TAVI, non-vitamin K antagonist oral anticoagulants (NOAC) or Vitamin K antagonists (VKA) are frequently switched to low-molecular weight heparin during diagnostic workup in patients with an indication for oral anticoagulation (mostly non-valvular atrial fibrillation) and resumed after the procedure. Several trials demonstrated that uninterrupted anticoagulation throughout cardiovascular procedures, e.g., catheter ablation for atrial fibrillation, was safe and associated with a lower risk for bleeding or stroke (32, 33). First non-randomized data suggest that the continuation of NOAC or VKA may also be safe and effective in patients undergoing TAVI without increased bleeding risk (34). A potential advantage of uninterrupted anticoagulation in these patients, a reduction in peri-procedural strokes, has yet to be shown.

### Procedural Anticoagulation and Heparin Reversal

TAVI procedures are performed after anticoagulation with unfractionated heparin to achieve a target activated clotting time of >250 s. At the end of the procedure, protamine is frequently given for heparin reversal to avoid bleeding complications. A recent non-randomized, single center analysis of 873 TAVI procedures found a significant reduction of the primary endpoint (all-cause mortality, major bleeding and life threatening bleeding), of 3.2% after heparin reversal with protamine compared to 8.7% in the control group, driven by less bleeding complications (35). Importantly, no elevated rates of ischemic events were found, However, the limited power and study design may not have been sufficient to adequately evaluate this essential aspect. Whether heparin reversal may increase the risk for ischemic events in patients with a high baseline stroke risk remains unclear at current. Further investigations are warranted to address this aspect and help to further tailor procedural management to the patients’ needs.

### Antithrombotic Strategies After TAVI

Following the procedure, sufficient antithrombotic therapies or oral anticoagulation aim to prevent ischemic events early and late after TAVI. However, in this vulnerable patient population, ischemic risk must be weighed against bleeding risk, particularly in the early postprocedural phase. Recently, several trials explored different therapeutic regimen in patients with and without an indication for oral anticoagulation due to atrial fibrillation, the latter of whom are at particular risk for cerebrovascular events during follow-up after TAVI.

In general, oral anticoagulation prevents embolism of fibrin-rich thrombi which mainly occur in areas of low shear stress (e.g., left atrial appendage) while antiplatelet therapy is thought to prevent platelet-rich thrombi developed in areas of high shear stress (e.g., transcatheter heart valve stent frame) (36). Accordingly, adding an antiplatelet agent to oral anticoagulation after TAVI may be beneficial to reduce ischemic events after TAVI. Interestingly, the following trials draw a different picture:

In the POPular TAVI trial aspirin alone was associated with a lower incidence of bleeding (hazard ratio [HR] 0.57; CI 0.42–0.77) and the composite of bleeding or thromboembolic events (HR 0.74; CI 0.57–0.95) at 1 year compared to aspirin plus clopidogrel administered for 3 months (37). In patients with an indication for long-term oral anticoagulation, the incidence of bleeding was lower with oral anticoagulation alone compared to oral anticoagulation and clopidogrel (HR 0.63; CI 0.43–0.90) (38). Importantly, no increased rates of ischemic events were observed in the monotherapy groups of either study arm. Interestingly, although most endpoint-related bleeding events were classified as non-procedural bleedings, Kaplan-Meier event curves diverge in the very early postprocedural period. Timing of initiation and loading dose of antithrombotic medication may have impacted these findings. The ongoing CLOE trial is designed like POPular TAVI but will enroll up to 4,000 patients to investigate the role of clopidogrel on top of aspirin or oral anticoagulation. These results might help us to define a tailored antithrombotic approach.

The ATLANTIS (39) and the ENVISAGE-TAVI AF (40) trials investigated the safety and efficacy of direct oral anticoagulants vs. vitamin K antagonists after TAVI in patients with non-valvular atrial fibrillation. In patients with successful TAVI, edoxaban was non-inferior to VKA for a composite primary outcome of adverse clinical events (death, myocardial infarction, ischemic stroke, systemic embolism, valve thrombosis, and major bleeding). However, the he incidence of major bleeding—mainly driven by gastrointestinal bleeding—was higher in the edoxaban group (hazard ratio 1.40, 95% CI 1.03–1.91) (40). Although superiority for the net clinical benefit was missed in the ATLANTIS trial comparing apixaban with standard of care (VKA or antiplatelet, depending on the indication for oral anticoagulation), event rates for the composite primary and safety bleeding endpoints were similar in both groups (41). The trial design comprising two strata additionally allowed for evaluation of apixaban therapy in patients without an indication for oral anticoagulation (39). However, in line with earlier data from the GALILEO trial (42), ATLANTIS demonstrated an unfavorable risk-benefit ratio for DOAC therapy compared to standard antiplatelets in patients without an indication for oral anticoagulation.

These results led to a de-escalation of antithrombotic regimen after TAVI in most heart teams and a corresponding update in the recent European guidelines on the management of valvular heart disease (43). However, several aspects, including the timing and initiation of antithrombotic therapy, remain unclear at current and deserve further investigation, particularly for the prevention of periprocedural stroke events.

## Conclusion

In summary, cerebrovascular events in patients undergoing TAVI remain a multifactorial phenomenon (36) with different predisposing factors according to symptom onset (18). Prevention should include procedural and periprocedural strategies. More randomized data are necessary to clarify open questions concerning the clinical benefit of CEP, anticoagulation and antithrombotic strategies in this setting.

## Author Contributions

Both authors listed have made a substantial, direct, and intellectual contribution to the work, and approved it for publication.

## Conflict of Interest

The authors declare that the research was conducted in the absence of any commercial or financial relationships that could be construed as a potential conflict of interest.

## Publisher’s Note

All claims expressed in this article are solely those of the authors and do not necessarily represent those of their affiliated organizations, or those of the publisher, the editors and the reviewers. Any product that may be evaluated in this article, or claim that may be made by its manufacturer, is not guaranteed or endorsed by the publisher.
